# Network analysis of ChIP-Seq data reveals key genes in prostate cancer

**DOI:** 10.1186/s40001-014-0047-7

**Published:** 2014-09-03

**Authors:** Yu Zhang, Zhen Huang, Zhiqiang Zhu, Jianwei Liu, Xin Zheng, Yuhai Zhang

**Affiliations:** Department of Urology, Beijing Friendship Hospital, Capital Medical University, Beijing, 100050 China; Department of Urology, Beijing You An Hospital, Capital Medical University, Beijing, 100069 China

**Keywords:** ChIP-Seq, Highly connected genes, Network analysis, Prostate cancer

## Abstract

**Background:**

Prostate cancer (PC) is the second most common cancer among men in the United States, and it imposes a considerable threat to human health. A deep understanding of its underlying molecular mechanisms is the premise for developing effective targeted therapies. Recently, deep transcriptional sequencing has been used as an effective genomic assay to obtain insights into diseases and may be helpful in the study of PC.

**Methods:**

In present study, ChIP-Seq data for PC and normal samples were compared, and differential peaks identified, based upon fold changes (with *P*-values calculated with *t*-tests). Annotations of these peaks were performed. Protein–protein interaction (PPI) network analysis was performed with BioGRID and constructed with Cytoscape, following which the highly connected genes were screened.

**Results:**

We obtained a total of 5,570 differential peaks, including 3,726 differentially enriched peaks in tumor samples and 1,844 differentially enriched peaks in normal samples. There were eight significant regions of the peaks. The intergenic region possessed the highest score (51%), followed by intronic (31%) and exonic (11%) regions. The analysis revealed the top 35 highly connected genes, which comprised 33 differential genes (such as *YWHAQ*, tyrosine 3-monooxygenase/tryptophan 5-monooxygenase activation protein and θ polypeptide) from ChIP-Seq data and 2 differential genes retrieved from the PPI network: *UBA52* (ubiquitin A-52 residue ribosomal protein fusion product 1) and *SUMO2* (SMT3 suppressor of mif two 3 homolog 2) .

**Conclusions:**

Our findings regarding potential PC-related genes increase the understanding of PC and provides direction for future research.

**Electronic supplementary material:**

The online version of this article (doi:10.1186/s40001-014-0047-7) contains supplementary material, which is available to authorized users.

## Background

Prostate cancer (PC) is the second most common cancer among men worldwide [1]. The most common symptoms are difficulty in urinating, erectile dysfunction and problems during sexual intercourse [2,3]. Genetic background contributes to PC risk, as suggested by associations with race, family and specific gene variants [4,5]. Many genes have been found to be involved in PC. For example, mutations in *BRCA1* (breast cancer 1, early onset) and *BRCA2* (breast cancer 2, early onset) are important risk factors for PC [6]. The authors of a previous article reported that *PTEN* (phosphatase and tensin homolog) deletions are related to tumor aggression in PC [7].

Determining how proteins interact with DNA is important to fully view many biological processes and disease states. The information thus derived can lead to a deeper understanding of tumor development. Chromatin immunoprecipitation sequencing (ChIP-Seq) is used to investigate interactions between chromatin-associated protein and DNA [8]. It provides the ability to identify the binding sites of any DNA-associated proteins [9]. CCCTC binding factor (CTCF) binds to three regularly spaced repeats of the core sequence CCCTC, and it is widely applied in ChIP-Seq [9,10].

ChIP-Seq analysis has recently been used to study PC. The results of these studies provide some meaningful ChIP-Seq data in comprehending the molecular mechanisms of androgen receptors (AR) in PC cells, which may be used to develop novel drugs [11,12]. Yu *et al*. [13] systematically mapped the genomic landscape of transcription factors and histone marks across multiple PC cell lines and tissues. ChIP-Seq has also been used to reveal direct binding of AR and ERG (v-ets avian erythroblastosis virus E26 oncogene homolog) to the promoter of glycine N-methyltransferase in VCaP (vertebral cancer of the prostate) cells [14]. There has been little study of the identification of PC-related genes by ChIP-Seq analysis.

Protein–protein interaction (PPI) networks provide valuable information in the understanding of cellular function and biological processes. With the tremendous increase in human protein interaction data, the PPI network approach is used to understand molecular mechanisms of disease, particularly to analyze cancer-related phenomena [15]. PPI networks also provide insights into distinct topological features of cancer genes [16]. Therefore, in the present study, we analyzed ChIP-Seq data derived from PC samples and normal controls, and differential peaks were screened out. Annotations were given for those peaks, and PPI network analysis was performed to identify critical genes related to PC.

## Methods

### Data source

Two Sequence Read Archive files, SRR513122 for normal and SRR513123 for tumor, were downloaded from National Center for Biotechnology Information Gene Expression Omnibus database with accession number [GEO:GSE38684] [17]. The original ChIP-Seq data were acquired from two normal primary prostate epithelial cells (PrEC cell lines) and three PC cell lines (LNCaP cell lines) by Bert *et al*. [17].

### Processing and alignment

Reads with low quality were first discarded. To prevent high-quality reads from being rejected during quality-filtering or assembly processes, we trimmed bases from poor-quality ends of reads [18]. ChIP-Seq reads were aligned to the human genome (hg19) using the Bowtie tool [19], which allows up to two mismatches in the alignment. Locations with one or more exact matches were kept for further analysis.

### Screening and annotation of differential peaks

Model-based analysis of ChIP-Seq (MACS) [20] was applied to identify peaks (read-enriched regions) from Sequence Alignment/Map (SAM) format files as well as differential peaks between cancer and normal samples. MACS is a command line tool designed to analyze ChIP-Seq data in eukaryotes, especially in mammals [21]. The parameters for calling peaks are as follows: (1) effective genome size = 2.70e + 09, (2) bandwidth = 300 bp and (3) *P*-value cutoff = 1.00e-10. To provide further functional context for the biological interpretation, peaks with fold enrichment scores above 2.95 were picked out and annotated with respect to known genomic sequence features (such as genes and transcripts) according to the hg19 refGenes data. Some peaks of various widths were assigned to a gene based on the closest transcription start sites (TSSs), then the locations of those peaks were extracted by MACS. DNA motifs were identified using MDSeqPos (*P* < 0.0001).

### Protein–protein interaction network analysis

Proteins (genes) work together to exert certain biological functions, with the proteins exhibiting more interactions with others likely to play more important roles in the whole process. Therefore, PPI network analysis of the genes obtained above was performed with BioGRID (Biological General Repository for Interaction Datasets) [22], which is a public database of physical and genetic interactions for all major model organism species (combined score above 0.4). This network was then constructed using Cytoscape [23], which is a software package available on the internet for biological network visualization, data integration and interaction network generation.

## Results

### Differential peaks between prostate cancer tissue samples and normal samples

**Figure 1:**
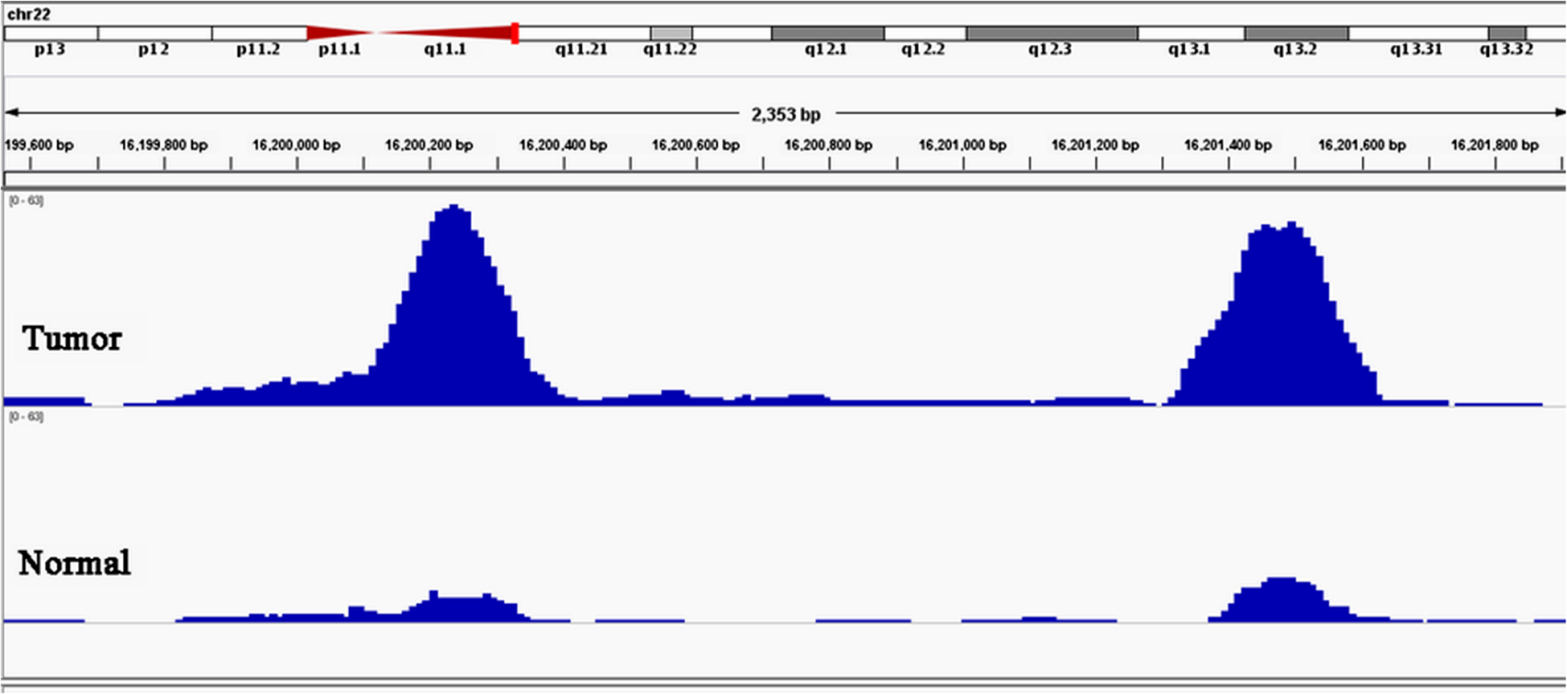
**Differential CCCTC binding factor binding peaks between the prostate cancer sample and the normal sample.** The chromosomal location is shown in the upper panel. Blue peaks represent CCCTC binding factor binding peaks in these regions.

A total of 5,570 differential peaks were found between the PC sample and normal sample (Additional file 1: Table S1 and Additional file 2: Table S2). Among these peaks, 3,726 peaks were differentially enriched in the PC sample and 1,844 peaks were differentially enriched in the normal sample. Two typical differential binding regions which were in the vicinity of *POTEH* (POTE ankyrin domain family, member H) are shown in Figure 1. We found that, in the tumor sample, both of these two peaks were significantly higher than those in the same positions of the normal sample, which may mean they are at the CTCF binding site in tumors. The widths of the two peaks in the tumor sample were approximately 200 to 300 bp.

### Annotation of differential peaks

**Figure 2:**
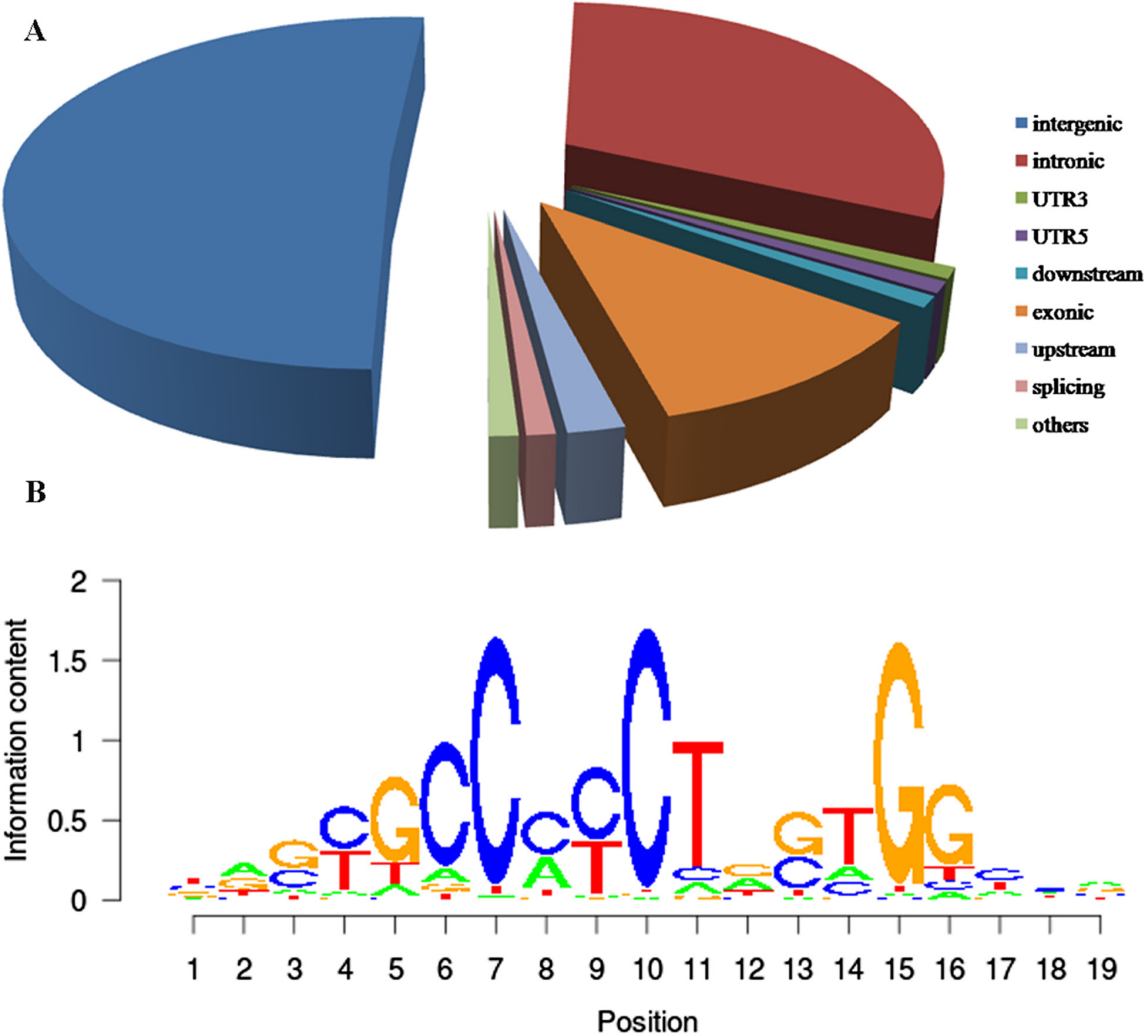
**The distribution regions and motifs with annotation of differential peaks. (A)** A pie chart of the peaks mapping to the splicing, upstream, exonic, downstream, 5′ untranslated region (UTR5), UTR3, intronic and intergenic genomic regions. **(B)** One of the top enriched DNA binding motifs.

Differential peak regions were annotated and their functional consequence on genes were examined (Additional file 3: Table S3). The most significantly regions of the peaks were shown in Figure 2A. The intergenic region was the highest with a score of 51%, followed by the intronic region (31%) and the exonic region (11%). Other regions of the peaks were upstream (2%), splicing (1%), the 5′ untranslated region (5′ UTR) (1%), 3′ UTR (1%) and downstream (1%). One of the top enriched DNA binding motifs is shown in Figure 2B. The − log_10_ (motif enrichment *P*-value) was 69.078.

### Protein–protein interaction network of peak-associated genes

**Figure 3:**
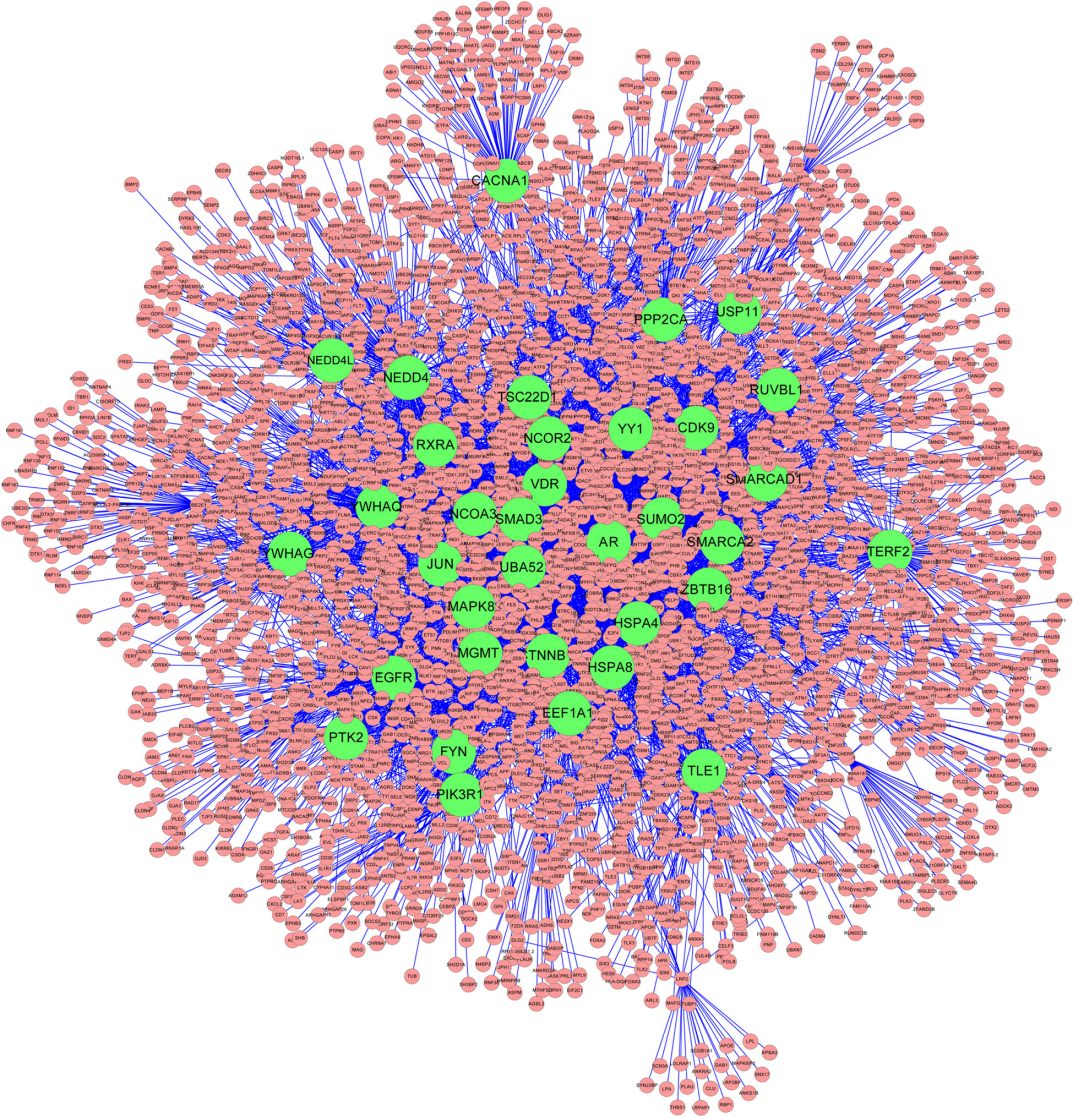
**The protein–protein interaction network of differential peak–related genes.** Nodes stand for genes or interacting genes. Edges indicate that some interaction exists in two nodes, such as the same pathways, biological processes or molecular functions. The interaction network was visualized with Cytoscape.

**Figure 4:**
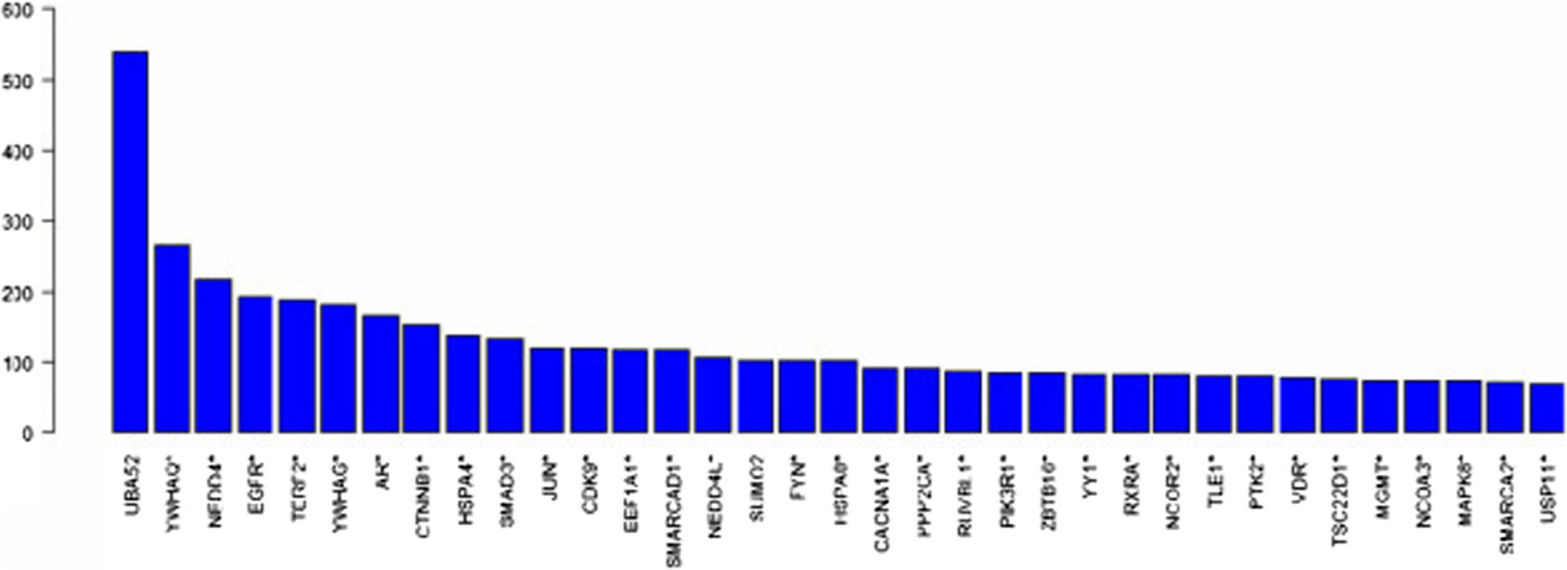
**Top 35 highly connected genes in the network of differential peak–related genes.** The ordinate represents the degree of protein. Genes marked with asterisks are differential peak–related genes that were identified from ChIP-Seq data.

The PPI network of peak-associated genes and some other interactional genes is shown in Figure 3 (*P* < 0.05). The network contains 4,920 protein nodes and 7,018 interaction edges. Genes closely related to others might have important roles in biological processes. Therefore, interactions between genes were calculated for each gene, and the top 35 genes were obtained (Figure 4). Among them, 33 differential genes were identified from the ChIP-Seq data, and 2 others were derived from the PPI network. Each of them had a neighbor number near or above 100. *UBA52* (ubiquitin A-52 residue ribosomal protein fusion product 1), with the highest weight (above 500), was a PPI network–associated gene. Next, the subsequent genes were identified from ChIP-Seq data, such as *YWHAQ* (tyrosine 3-monooxygenase/tryptophan 5-monooxygenase activation protein, θ polypeptide), *NEDD4* (neural precursor cell expressed, developmentally downregulated 4, E3 ubiquitin protein ligase) and *EGFR* (epidermal growth factor receptor). Some other genes, including *AR* (androgen receptor), *HAPA4* (heat shock 70 kDa protein 4), *CDK9* (cyclin-dependent kinase 9) and *SUMO2* (SMT3 suppressor of mif two 3 homolog 2), may be PC-related.

## Discussion

Unlike sequence-specific transcription activators such as ER and p53, CTCF binding sites are ubiquitously and universally present throughout the genome, and their chromosomal distribution is strongly correlated with genes [24]. Bert *et al*. used these data to identify the relationship between long-range epigenetic activation and domain gene deregulation, which is quite different from our results [17]. We used MACS was in the present study and found 5,570 differential peaks comprising 3,726 differentially enriched peaks in the PC sample and 1,844 differentially enriched peaks in the normal sample. According to the annotation results (Figure 2), CTCF binding sites were scattered in chromosomes. About one-half of the CTCF binding sites flanked genes. In addition, some differentially expression genes were obtained with the PPI network analysis.

Figure 4 shows the top 35 highly connected genes in a network of differential genes. Some of them are biomarkers of PC. *AR* had the sixth most interactors in the network, and the peak of it was enriched in the intergenic region of chromosome X, which is in accordance with the results reported by Taslim *et al*. [25]. The AR signaling axis plays a critical role in PC development and progression [26]. In a previous study, researchers discovered that AR is highly expressed in PC and that it may inhibit PC progression by suppressing AR expression and activity [27]. An international patent has been filed for the use of AR variants as biomarkers and therapeutic targets in advanced PC [28].

CDK9 is a regulator of cell cycles. The peaks of *CDK9* were aligned upstream of chromosome 9. Shore *et al.* showed that one isoform of CDK9 was transcribed from an alternative upstream promoter [29]. Gordon *et al*. found that CDK9 regulated AR promoter selectivity and cell growth through serine 81 phosphorylation [30]. The combined inhibition of Cdk9 and Akt can be utilized to induce apoptosis of metastatic PC cells [31].

EGFR is also an important player in the PPI network. The position of differential peaks annotation was exonic at chromosome 7. EGFR is the cell-surface receptor for members of the epidermal growth factor family of extracellular protein ligands [32]. EGFR overexpression may serve as a reasonable target for therapeutic intervention in this otherwise difficult-to-treat subset of PC patients [33]. The progression of PC is accompanied by the overexpression of EGFR in a very large majority of cases, suggesting that it may play a crucial role in PC [34].

*NEDD4*, whose peak annotation was located in the intergenic region of chromosome 15, was in the center of the PPI network. It is a proto-oncogenic ubiquitin ligase for PTEN, and its upregulation is found in many human cancers [35]. Farooqi *et al*. reported that *SMURF* and *NEDD4* interference offers therapeutic potential in chaperoning genome integrity [36].

The peak of *HSPA4* (heat shock protein 70 (HSP70)) was located in the intergenic region of chromosome 5. HSPA4 has been implicated in PC [37,38], and it may also be differentially regulated according to our analysis. Kottke *et al*. found that induction of hsp70-mediated Th17 autoimmunity can be exploited in immunotherapy for metastatic PC [39].

*SUMO2* was also included in the list. In previous studies, investigators have discovered the role of desumoylation in the development of PC [40–42]. Yang *et al*. reported that small ubiquitin-like modifier isoforms 1, 2 and 3 are activated in human astrocytic brain tumors and are required for glioblastoma cell survival. Therefore, we speculated that SUMO2 might play a similar role in PC.

*POTEH*, located on chromosomes 21, may also be a candidate for the immunotherapy of PC [43], which is recorded in GeneCards as a PC-related gene. A previous study showed that *POTEH* is expressed highly in PC, but is limited in benign tissues [44].

## Conclusions

Overall, we identified a number of key genes related to PC by analyzing ChIP-Seq data in the present study. These genes include *AR*, *CDK9*, *EGFR*, *NEDD4*, *HSPA4* and *SUMO4*, and about one-half of them were located in the intergenic regions of chromosomes with differential peak annotations. These genes may help enhance the understanding of PC and also provides direction for future research. Future research is needed to define their roles in detail and subsequently develop effective molecular target therapies for PC.

## Additional files

Additional file 1: Table S1. Peaks were significantly enriched in tumor sample. Additional file 2: Table S2. Peaks were significantly enriched in normal sample. Additional file 3: Table S3. Differential peaks annotation.
